# Patient and family views of team functioning in primary healthcare teams with nurse practitioners: a survey of patient-reported experience and outcomes

**DOI:** 10.1186/s12875-021-01406-y

**Published:** 2021-04-19

**Authors:** Kelley Kilpatrick, Eric Tchouaket, Nicolas Fernandez, Mira Jabbour, Carl-Ardy Dubois, Lysane Paquette, Véronique Landry, Nathalie Gauthier, Marie-Dominique Beaulieu

**Affiliations:** 1 Susan E. French Chair in Nursing Research and Innovative Practice, Ingram School of Nursing, Faculty of Medicine and Health Sciences, McGill University, Montréal, Québec Canada; 2 Department of Nursing, Université du Québec en Outaouais (UQO), St-Jérôme Campus, Saint-Jérôme, Québec Canada; 3 Centre for Pedagogy Applied to the Health Sciences, Department of Family Medicine and Emergency Medicine, Faculty of Medicine, Université de Montréal, Montréal, Québec Canada; 4 Centre intégré universitaire de santé et de services sociaux de l’Est-de-l’Île-de-Montréal, Maisonneuve-Rosemont Hospital Site, Montréal, Québec Canada; 5 Department of Management, Evaluation and Health Policy, School of Public Health, Université de Montréal, Montréal, Québec Canada; 6 Faculty of Nursing, Université de Montréal, Montréal, Québec Canada; 7 Nursing and Physical Health Directorate, Centre intégré universitaire de santé et de services sociaux de La Capitale-Nationale, Québec, Québec Canada; 8 Department of Family Medicine and Emergency Medicine, Faculty of Medicine, Université de Montréal, Montréal, Québec Canada

**Keywords:** Mediation, Nurse practitioner, Patient-reported experience measure, Patient-reported outcome measure, Perceptions of team effectiveness, Process, Team functioning

## Abstract

**Background:**

Nurse practitioners (NPs) have been added to primary healthcare teams to improve access to care. Team processes, including communication and decision-making, explicate how patients and families view team functioning. Yet, important gaps exist in our understanding of patient-reported experience and outcomes at the level of the healthcare team. We aimed to examine the influence of individual, team, and organizational characteristics, and role clarity on outcomes of care mediated by team processes in primary healthcare teams that include NPs.

**Methods:**

A cross-sectional survey across six sites representing practices with NPs in Québec, Canada, was conducted between March 2018 and April 2019 as part of a multiple-case study. Patients and families (*n *= 485; response rate: 53%) completed a validated questionnaire, which included a patient-reported experience measure (PREM) and a patient-reported outcome measure (PROM) of team functioning (Cronbach alpha: 0.771 (PROM) to 0.877 (PREM)). We performed logistic regression and mediation analyses to examine relationships between the individual, team, and organizational characteristics, role clarity, and outcomes of care mediated by team processes.

**Results:**

Patients and families expressed positive perceptions of team functioning (mean 4.97/6 [SD 0.68]) and outcomes of care (5.08/6 [0.74]). Also, high team processes (adjusted odds ratio [AOR] 14.92 [95% CI 8.11 to 27.44]) was a significant predictor of high outcomes of care. Role clarity (indirect effect coefficient ab = 6.48 [95% CI 3.79 to 9.56]), living in an urban area (-1.32 [-2.59 to -0.13]), patient as respondent (-1.43 [-2.80 to -0.14]), and income (1.73 [0.14 to 3.45]) were significant predictors of outcomes of care mediated by team processes.

**Conclusions:**

This study provides key insights on how primary healthcare teams with NPs contribute to team functioning, using a validated instrument consistent with a conceptual framework. Results highlight that high role clarity, living in a non urban area, family as respondent, and adequate income were significant predictors of high outcomes of care mediated by high team processes. Additional research is needed to compare teams with and without NPs in different settings, to further explicate the relationships identified in our study.

## Background

There is worldwide interest in measuring patient experience and patient-reported outcomes [1]. Currently, patient-reported experience measures (PREMs) report on what occurs and how events unfold during a healthcare visit from the patient’s perspective [1], whereas patient-reported outcome measures (PROMs) examine the impact of health conditions and the effectiveness of care from the patient’s perspective. Systems measuring PROMs such as PROMIS© have grown phenomenally, with over 300 validated instruments currently in their database [2]. However, in their systematic review of PREMs, Bull et al. [1] found that these instruments remained focussed on episodic care in hospitals and interactions at the patient-provider level specific to a condition or a setting. No measures were identified at the healthcare team level. In another systematic review of 321 PROMs in primary care, Murphy et al. [3] found limited consensus on appropriate PROMs to use to capture the overall outcome of a primary care visit. Thus, to better understand patient experience and outcomes in primary healthcare, it is essential to capture team-level processes.

Team processes explicate patient and family perceptions about team effectiveness (PTE) and how teams function, and include decision-making, clear communication, care coordination, cohesion, problem-solving, and a focus on the needs of patients and families [4]. Furthermore, processes explain how situations unfold over time in response to events in the surrounding context [5]. Researchers have likened team processes to a black box because it is difficult to capture the jointly created rules, complex interactions, and relationships that occur between individuals [5]. In 2014, Strasser et al. [6] noted that measures of team functioning sufficiently sensitive to identify changes in patient outcomes were only just emerging [6]. Such measurement of team functioning from the patient and family perspective has been a critical challenge because of the human and financial resources required and the lack of validated instruments available [4, 7].

Evaluating how teams function is important because poor team functioning can lead to patient harm [8]. In a recent report for the Organisation for Economic Co-operation and Development, Auraanen et al. [9] found that lapses in primary care are common and represent approximately half of the global burden of patient harm. These authors argued that integrated information systems, patient involvement in measurement, and increased teamwork were essential conditions to improve safety in primary care [9]. Although performance of primary healthcare teams and patient experience have been examined using *system*-level indicators, including access to care, wait times, and rates of cancer screening [10], as a measure of performance, *individual* assessments must be aggregated to the team, organizational or system level to then be able to track results locally, nationally, and internationally [10].

Given the growing pressures to improve team performance, different professionals have been added to primary healthcare teams to increase access to safe patient care [11]. In primary healthcare, Hofhuis et al. [11] found that team performance was influenced by how frequently team members interacted with each other. Nurses can make important contributions to team member interaction and access to care as they make up the largest portion of the global healthcare workforce [12]. Making the best use of nurse roles is critical to support efforts to reach the World Health Organization’s [12] Sustainable Development Goals centered on universal access to healthcare. Nurse practitioners (NPs), recognized as advanced practice nurses, have developed globally to respond to the inadequate supply of primary care providers [13]. However, NP role implementation has been unequal internationally [14]. Over half of the studies included in a scoping review of the international literature on barriers and facilitators of NP role implementation were conducted in North America [14]. In the United States, where NPs were implemented in the 1960s [14], their numbers have increased exponentially and more than doubled between 2010 and 2017, reaching a total number of 190,000 [15]. Other countries and jurisdictions within countries have implemented NPs in primary care at a much slower rate [14].

NPs are registered nurses trained at the graduate level who have acquired in-depth clinical knowledge, skills, and decision-making autonomy for expanded practice [16]. Evidence from systematic reviews of effectiveness and cost-effectiveness have highlighted that NPs in primary care improve access to and the quality of care for patients and families, particularly for vulnerable patients or those living in underserviced areas [17]. Furthermore, NPs reduce healthcare costs when they work to their optimal scope of practice [18]. In long-term care, primary healthcare NPs reduce polypharmacy rates, the number of unnecessary medications, admissions to acute care, transfers to the emergency department, and costs [19, 20]. Nevertheless, NP consultation times may be longer than physician consultation times, particularly when scope of practice regulations are too restrictive [17, 18]. Even so, researchers [21] have argued that NPs’ holistic decision-making and supportive communication may contribute to longer consultation times and improve patient experience of care. In addition to holistic decision-making and supportive communication, teamwork and patient engagement are essential in the delivery of safe and effective care [22].

Although patients and families consistently report that they are satisfied with care provided by primary healthcare teams with NPs [18], there are important gaps in our understanding of how these teams function because research so far has focussed on individual-level interactions with providers rather than on micro-level processes in healthcare teams [4, 23]. There is a lack of research measuring team processes rather than outcomes of teamwork [24], and little is known about the relationship between individual, team and organizational characteristics, and team processes on outcomes [25]. Furthermore, to understand patient experience and how teams with NPs function in the context of patient-centered care [23], it is essential to include patients and families. This paper describes the views of patients and families of how their healthcare teams with NPs function. More specifically, in primary healthcare teams with NPs, we aim to examine the influence of individual, team and organizational characteristics, and role clarity on outcomes of care (PROM) mediated by team processes (PREM). Because NP roles in primary care were emerging in Québec at the time of the study, we aimed to describe how teams with NPs influence team functioning.

## Conceptual framework

**Fig. 1:**
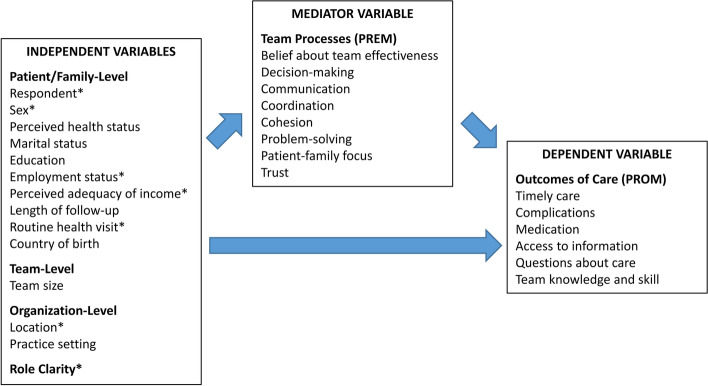
Relationships between the independent variables, team processes, and outcomes of care. * Independent variable presenting a statistically significant relationship in at least one of the three models

We adapted the conceptual framework describing perceptions of team effectiveness (PTE) in healthcare teams with NPs by Kilpatrick et al. (2013) [26] (See Fig. 1). The framework was based on the structures-processes-outcomes model. The structural dimension includes factors at the patient, team and organizational levels. Team processes include trust and PTE (e.g., communication, care coordination) with a focus on the needs of patients and families (PREM). Role clarity, defined as understanding one’s role and the role of others in the team, has been identified as a critical factor to improve team functioning [27, 28]. The lack of role clarity can jeopardize teamwork. Outcomes of care are defined as the results of care provided by the healthcare team (PROM). The adapted framework illustrates direct relationships between the patient, team and organizational characteristics, role clarity, and outcomes of care and indirect effects mediated by team processes.

## Methods

We conducted a cross-sectional survey across six sites as part of a mixed methods multiple-case study in Québec, Canada. Québec is the second largest province in Canada in terms of population. NP roles in primary care were implemented in 2007 [16] in Québec. NPs in primary care practice in offices, home care, and long-term care [16]. We purposefully selected sites to represent practices with NPs in Québec across a range of characteristics [17], including rural and urban settings, type of practice, and team size.

### Data collection and recruitment

Data were collected as part of a larger study examining how teams with NPs optimize roles of members of the healthcare team, including patients and families (2016 to 2019). The survey was administered between March 2018 and April 2019, following research ethics board approval. The minimum sample size of 400 was required to obtain significant results from a logistic regression with 6 significant independent variables (IVs) in the final model [29].

Each site included between one to three NPs, and each NP worked with one healthcare team in the organization. Respondents in four of the six sites identified their location as rural. Patients needed to be cared for by a healthcare team with an NP. Family included persons significant to the patient who were involved in their care. Participants were randomly selected from the NPs’ caseload using a computer-generated random sequence. We recruited 200 patients and families where the NP’s caseload included at least 200 patients. The three NPs working in home care and long-term care had a caseload of less than 200 patients, and all the patients in their caseload were recruited. We used Dillman’s method with four mailing cycles [30]. Participants could complete the questionnaire on paper or on-line. A self-addressed stamped return envelope was included. A total of 982 questionnaires were mailed out, 75 were returned undelivered, and 485 questionnaires were completed (response rate: 53%). Questionnaires completed on-line represented 28% (*n *= 134) of responses. A small voucher incentive ($5-gift card at a national coffee house) was included with the first mailing as a thank you gesture.

### Instrument

The Patient-PTE Questionnaire is a 43-item self-report instrument that measures how teams function. It is available in English and in French and takes approximately 10 min to complete [4]. Responses range from 1 (strongly disagree) to 6 (strongly agree) on a scale with no neutral point. Higher scores indicate improved PTE and team functioning. One question (Q. 14) specifically examines beliefs about team effectiveness (BE). One question (Q. 8) describes the NP role and asks respondents if their team includes an NP. One open-ended question (Q. 43) gathers additional comments. Psychometric testing (*n *= 355) of the instrument included construct validity using known groups with additional testing in teams with physiotherapists in extended roles [4]. Differences were identified by clinical specialty, patient education, length of follow-up, and reason of the health visit. Reliability was assessed using Cronbach α with values ranging from 0.76 to 0.94. Rho coefficients (rs) for processes included in the Team Processes subscale ranged from 0.55 to 0.79 (*p* < 0.001) indicating that these processes were highly correlated with BE. Responsiveness was assessed and differences were noted in low and high functioning teams (*p* < 0.001). Individual results can be aggregated to the team, organization, or system level. The questionnaire measures teamwork by including key dimensions of team functioning consistent with our conceptual framework based on the structures-processes-outcomes model and by situating patients and families as members of the healthcare team. Using the current sample, reliability was assessed for the 15-item Team Processes (PREM) and Outcomes of Care (PROM) subscales using Cronbach α. Values ranged from 0.771 (PROM) to 0.877 (PREM).

### Operational description of variables

All the variables needed for the analysis were included in the questionnaire and consistent with our conceptual framework. Given that age is not specific to team care in primary care, we included age as a descriptive variable rather than a variable in the predictive models.

### Dependant variable

The Outcomes of Care subscale represents a PROM of team functioning. Six items measure timely care, promptly dealing with potential or actual complications, medication, access to information, answers to questions about care, and knowledge and skill of the healthcare team.

### Independent variables

Individual variables included *patient and family characteristics* with type of respondent (patient, family), age (years), sex (male/female), perceived health status (low: poor, fair; high: good, very good, excellent), marital status (living with a partner: yes/no), education (completed high school: yes/no), employment status (not employed/employed), financial status (perceived income adequate/ inadequate), length of follow-up (less than 24 months/ greater than 24 months), routine health visit (yes/no), country of birth (Canada: yes/Other: no).

Team characteristics included *healthcare team size* with small (less than 5 team members), medium (5 to 10 team members), large (more than 10 team members). Team size was dichotomized as small/ medium-large.

Organizational characteristics were measured using two items. *Healthcare setting* included 15 possible response options in the questionnaire. Survey responses were grouped into primary care and home care/long-term care. *Location* including urban, rural, remote and rurban, was dichotomized as urban/non urban.

*Role clarity* was measured using two items (i.e., how well roles are defined in teams, how work is divided among team members).

### Mediator variable

*Perception of Team Effectiveness* includes 15 items: decision-making (information is shared, ideas are valued), communication (plan of care is communicated, health record is up-to-date, flow of information), care coordination (next steps in care plan, care adjusted to change in patient’s condition, care well organized), cohesion (working together), problem-solving (differences of opinion are respected), patient and family focus (patient/family has a role in the team, contribution is valued, working with family), trust (trust in team), and belief about team effectiveness (team is effective).

### Analysis

Logistic regression and mediation analyses were used to examine the influence of the IVs (i.e., individual, team, and organizational characteristics) and role clarity on outcomes of care mediated by team processes. We then examined the influence of team processes on outcomes of care, the dependent variable (DV) [31, 32]. Analyses were completed with IBM Statistical Package for Social Sciences version 27 (2020) [33] and STATA version 13 (2013) [34]. The 5% threshold was used to determine significance. Negatively worded items were reverse-coded prior to analysis. As described above, dichotomous variables were created for all the independent variables in the model. Numeric responses from 1 to 4 were recoded as low, and responses 5 and 6 were recoded as high. Descriptive statistics (number and proportion) were generated. Bi-variate analyses were conducted to examine significant relationships between the independent variables, team processes, and outcomes of care. Multicolinearity between the individual characteristics was examined [31]. Perceived health status and education were highly correlated (*p < *0.001 with other individual characteristics and were not included in the models. Unadjusted odds ratios (UORs) were estimated. Finally, we performed three multivariate binary logistic regression and mediation analyses to identify in primary healthcare teams with NPs the (i) IVs (i.e., individual, team and organizational characteristics, and role clarity) that influence team processes (Model 1); (ii) IVs that influence outcomes of care (Model 2); (iii) influence of the IVs and team processes on outcomes of care (Model 3). Coefficients for the indirect effects were calculated using unstandardized variables to identify the potential mediator effect of team processes [32]. The indirect effect (mediation) of team processes was considered when the effect of X on Y was weaker in Model 3 than in Model 2. This was considered complete indirect effect if X no longer had an effect on Y when the effect of the mediator was controlled (Model 3). Sensitivity analyses were performed to examine the indirect effect using Sobel test, Aroian test, and Goodman test [32, 35, 36]. As proposed by Rijnhart et al. (2019) [32], we used the following equations:

1 $$\mathrm{M}={\mathrm{i}}_2+\mathrm{aX}$$

2 $$\mathrm{Y}={\mathrm{i}}_1+\mathrm{cX}$$

3 $$\mathrm{Y}={\mathrm{i}}_3+\mathrm{c}'\mathrm{X}+\mathrm{bM}$$

Where: X represents the characteristics and role clarity (IV); Y represents the outcome (PROM); M represents the mediator (PREM); $i$ represent the intercepts; aX represents the slope of each independent variable; bM represents the slope of the mediator; cX represents the slope of outcome; and c’X represents the slope of outcome (when the IV and PREM are also a predictor of the PROM); ab represents indirect effect.

The 95% confidence interval (95% CI) of the indirect effect *ab* was determined by the set of values between the 2.5th percentile and the 97.5th percentile of the distribution of ab obtained by the hierarchical Bayesian method (simulation). Using the computational program developed by Falk and Biesanz (2016) [37] the confidence interval was calculated.

The variables that were included in the final model were kept because of their identified conceptual significance. We did not impute data. All the patients and families with at least one missing datum were removed during the multivariate analysis. The Stepwise procedure was used for the selection of variables retained in the models (using Likelihood Ratio) [38]. The Hosmer–Lemeshow statistic allowed us to determine the quality of the models [31].

### Patient and public involvement

Our research team's expert patient (NF) was involved in all phases of the study: development and validation of the Patient-PTE questionnaire, study conception and design, acquisition and analysis of study data, and dissemination.

## Results

Four hundred and eighty-five respondents completed the survey. Of these, 391 participants (81.0%) had complete datasets that were used for the logistic regression analysis. Sensitivity analysis allowed us to determine that there were no significant differences between the respondents who were included and those who were excluded from the analysis. The descriptive characteristics of our sample and mean values for team processes and outcomes of care are presented in Tables 1, 2, 3. More specifically, respondents were primarily patients (66.7%). Most respondents were followed by teams in primary care (87.6%) with less than five members (57.8%). Individual characteristics included female sex (73.1%), aged 15 to 64 (75.2%), being married/living with a partner (71.9%), completed high school education (88.2%), good to excellent perceived health status (90.1%), being employed (60.9%), adequate perceived income (79.6%), and located in an urban area (55.9%).

**Table 1:** Characteristics of respondents (*n *= 485)

	*n* (%)	Mean	SD	Minimum	Maximum
Patient variables					
Respondent					
Family	154 (33.4)				
Patient	309 (66.7)				
Age		50.46	17.33	15	96
Sex					
Male	127 (26.9)				
Female	345 (73.1)				
Perceived health status					
Low	47 (9.9)				
High	429 (90.1)				
Marital status (living with a partner)					
No	129 (28.1)				
Yes	330 (71.9)				
High school education completed					
No	55 (11.8)				
Yes	411 (88.2)				
Employment status					
Not employed	179 (39.1)				
Employed	279 (60.9)				
Perceived income					
Inadequate	96 (20.4)				
Adequate	374 (79.6)				
Length of follow-up					
> 24 months	363 (78.9)				
≤ 24 months	97 (21.1)				
Routine health visit					
No	34 (7.3)				
Yes	431 (92.7)				
Country of birth (Canada)					
No	50 (10.7)				
Yes	419 (89.3)				
Team variable					
Team size					
Medium/large (≥ 5 members)	195 (42.2)				
Small (< 5 members)	267 (57.8)				
Organizational variables					
Location					
Non urban	211 (44.1)				
Urban	268 (55.9)				
Practice setting					
Home care, long-term care	60 (12.4)				
Primary care	425 (87.6)				
Role clarity		5.28	0.79	1.00	6.00
Low	54 (11.6)				
High	413 (88.4)

**Table 2:** Description of Team Processes (*n *= 485)

	*n* (%)	Mean	SD	Minimum	Maximum
Team processes (PREM)		4.97	0.68	1.67	6.00
Low	207 (44)				
High	263 (56)				
Belief about team effectiveness (BE)		5.47	0.82	1.00	6.00
Low	30 (6.5)				
High	433 (93.5)				
Decision-making		5.31	0.81	1.00	6.00
Low	73 (15.7)				
High	392 (84.3)				
Communication		4.67	0.84	1.00	6.00
Low	268 (58.1)				
High	193 (41.9)				
Coordination		5.24	0.83	1.00	6.00
Low	92 (19.8)				
High	373 (80.2)				
Cohesion		5.31	0.85	1.00	6.00
Low	51 (11.0)				
High	411 (89.0)				
Problem-solving		5.17	0.81	1.00	6.00
Low	69 (16.1)				
High	359 (83.9)				
Patient-family focus		4.27	1.17	1.00	6.00
Low	296 (64.5)				
High	163 (35.5)				
Trust		5.40	0.90	1.00	6.00
Low	39 (8.4)				
High	426 (91.6)

**Table 3:** Description of Outcomes of Care (*n *= 485)

	n (%)	Mean	SD	Minimum	Maximum
Outcomes of care (PROM)		5.08	0.74	2.17	6.00
Low	151 (32.3)				
High	317 (67.7)				
Timely care		5.05	1.05	1.00	6.00
Low	95 (20.6)				
High	367 (79.4)				
Complications		5.23	0.90	2.00	6.00
Low	61 (13.6)				
High	389 (86.4)				
Medication		4.72	1.41	1.00	6.00
Low	116 (25.6)				
High	337 (74.4)				
Access to information		4.97	1.07	1.00	6.00
Low	99 (22.3)				
High	345 (77.7)				
Questions about care		5.10	1.08	1.00	6.00
Low	76 (16.6)				
High	382 (83.4)				
Team knowledge and skill		5.42	0.79	1.00	6.00
Low	36 (7.8)				
High	427 (88.0)				

*PREM* Patient-reported experience measure, *PROM* Patient-reported outcome measure

Overall, the mean value for team process was 4.97/6 (SD 0.68), with a range of 4.27/6 (SD 1.17) for patient-family focus to 5.47/6 (SD 0.82) for BE. The mean for role clarity was 5.28/6 (SD 0.79) and outcomes of care was 5.08/6 (SD 0.74).

Three models were developed to address the study aim. Each model is shown below with unadjusted odds ratios presented in Table 4 and adjusted odds ratios presented in Table 5.

**Table 4:** Unadjusted odds ratios for the relationships between the independent variables, team processes, and outcomes of care

Variables	Team processes (PREM)		Outcomes of care (PROM)	
	Unadjusted odds ratio (95% CI)	*p* value	Unadjusted odds ratio (95% CI)	*p* value
Patient variables				
Respondent				
Family	Reference	.	Reference	.
Patient	0.71 (0.48–1.05)	0.089	1.13 (0.74–1.71)	0.569
Sex				
Male	Reference	.	Reference	.
Female	1.34 (0.88–2.02)	0.169	1.36 (0.88–2.08)	0.165
Perceived health status				
Low	Reference	.	Reference	.
High	2.22 (1.92–4.12)	0.012	3.25 (1.76–6.01)	< 0.0001
Marital status (living with a partner)				
No	Reference	.	Reference	.
Yes	1.05 (0.69–1.58)	0.833	1.13 (0.73–1.75)	0.590
High school education completed				
No	Reference	.	Reference	.
Yes	1.22 (0.69–2.15)	0.499	1.40 (0.78–2.51)	0.262
Employment status				
Not employed	Reference	.	Reference	.
Employed	0.88 (0.60–1.29)	0.507	1.53 (1.02–2.29)	0.040
Perceived income				
Inadequate	Reference	.	Reference	.
Adequate	1.80 (1.14–2.83)	0.012	1.47 (0.92–2.34)	0.110
Length of follow-up				
> 24 months	Reference	.	Reference	.
≤ 24 months	1.13 (0.71–1.79)	0.620	0.78 (0.48–1.25)	0.297
Routine health visit				
No	Reference	.	Reference	.
Yes	1.45 (0.72–2.96)	0.301	3.63 (1.75–7.52)	0.001
Country of birth (Canada)				
No	Reference	.	Reference	.
Yes	0.76 (0.41–1.42)	0.389	1.32 (0.71–2.47)	0.381
Role clarity				
Low	Reference	.	Reference	.
High	10.79 (4.76–24.47)	< 0.0001	5.36 (2.89–9.95)	< 0.0001
Team variable				
Team size				
Medium/large (≥ 5 members)	Reference	.	Reference	.
Small (< 5 members)	1.35 (0.93–1.96)	0.120	1.30 (0.87–1.94)	0.194
Organizational variables				
Location				
Non urban	Reference	.	Reference	.
Urban	0.66 (0.46–0.96)	0.027	1.32 (0.90–1.95)	0.160
Practice setting				
Home care, long–term care	Reference	.	Reference	.
Primary care	1.54 (0.89–2.67)	0.125	1.68 (0.95–2.97)	0.073
Team processes (PREM)				
Low	.	.	Reference	.
High	.	.	11.81 (7.34–19.01)	< 0.0001
Outcomes of care (PROM)				
Low	Reference	.	.	.
High	11.81 (7.34–19.01)	< 0.0001	.	.

*PREM* Patient-reported experience measure, *PROM *Patient-reported outcome measure

**Table 5:** Adjusted odds ratios for the relationships between the independent variables, team processes, and outcomes of care

Variables	Model 1^a^			Model 2^b^			Model 3^c^		
	Coefficient	Adjusted odds ratio (95% CI)	*p* value	Coefficient	Adjusted odds ratio (95% CI)	*p* value	Coefficient	Adjusted odds ratio (95% CI)	*p* value
Team processes (PREM)									
Low	Reference	.	.	.	.	.	Reference	.	.
High	.	.	.	.	.	.	2.70	14.92 (8.11–27.44)	< 0.0001
Role clarity									
Low	Reference	.	.	Reference	.	.	Reference	.	.
High	2.40	11.07 (4.45–27.55)	< 0.0001	1.98	7.24 (3.52–14.89)	< 0.0001	1.11	3.02 (1.36–6.73)	0.007
Respondent									
Family	Reference	.	.	Reference	.	.	Reference	.	.
Patient	-0.53	0.59 (0.37–0.95)	0.029	0.14	1.15 (0.67–1.95)	0.615	0.59	1.81 (1.02–3.22)	0.043
Sex									
Male	Reference	.	.	Reference	.	.	Reference	.	.
Female	0.36	1.43 (0.88–2.31)	0.145	0.52	1.68 (1.03–2.75)	0.040	0.47	1.60 (0.89–2.88)	0.114
Perceived income									
Inadequate	Reference	.	.	Reference	.	.	Reference	.	.
Adequate	0.64	1.90 (1.06–3.42)	0.032	0.48	1.62 (0.90–2.91)	0.109	-0.05	0.95 (0.45–2.01)	0.891
Location									
Non urban	Reference	.	.	Reference	.	.	Reference	.	.
Urban	-0.49	0.62 (0.40–0.95)	0.029	0.35	1.42 (0.90–2.26)	0.134	0.82	2.27 (1.31–3.95)	0.004
Employment status									
Not employed	Reference	.	.	Reference	.	.	Reference	.	.
Employed	-0.44	0.64 (0.40–1.03)	0.064	0.13	1.14 (0.69–1.89)	0.613	0.65	1.92 (1.10–3.36)	0.021
Routine health visit									
No	Reference	.	.	Reference	.	.	Reference	.	.
Yes	0.41	1.50 (0.68–3.31	0.312	1.32	3.75 (1.73–8.14)	0.001	1.59	4.90 (1.96–12.22)	0.001

^a^Model 1: Dependent variable: **Team processes.** Independent variables: Role clarity, Team size, Respondent, Perceived income, Location, Practice setting, Employment status, Marital status, Sex, Length of follow-up, Routine health visit and Country of birth

Hosmer–Lemeshow: Khi2 = 3.373; ddl = 7; *p* value 0.848. Backward stepwise likelihood

^b^Model 2: Dependent variable: **Outcomes of care**. Independent variables: Role clarity, Team size, Respondent, Perceived income, Location, Practice setting, Employment status, Marital status, Sex, Length of follow-up, Routine health visit and Country of birth

Hosmer–Lemeshow: Khi2 = 0.018; ddl = 2; *p* value 0.991. Backward stepwise likelihood

^c^Model 3: Full model Dependent variable: **Outcomes of care**. Independent variables: Team processes, Role clarity, Team size, Respondent, Perceived income, Location, Practice setting, Employment status, Marital status, Sex, Length of follow-up, Routine health visit and Country of birth

Hosmer–Lemeshow: Khi2 = 8.632; ddl = 8; *p* value 0.374. Backward stepwise likelihood

*PREM* Patient-reported experience measure, *PROM* patient-reported outcome measure

Model 1 examined the independent variables that predict team processes. Role clarity (UOR 10.79 [95% CI 4.76 to 24.47]; adjusted odds ratio [AOR] 11.07 [95% CI 4.45 to 27.55]), living in an urban area (UOR 0.66 [0.46 to 0.96]; AOR 0.62 [0.40 to 0.95]), patient as respondent (UOR 0.71 [0.48 to 1.05]; AOR 0.59 [0.37 to 0.95]) and level of perceived income (UOR 1.80 [1.14 to 2.83]; AOR 1.90 [1.06 to 3.42]) predicted team processes.

Model 2 examined the independent variables that predict outcomes of care. High role clarity (UOR 5.36 [95% CI 2.89 to 9.95]; AOR 7.24 [95% CI 3.52 to 14.89]), sex (UOR 1.36 [0.88 to 2.08]; AOR 1.68 [1.03 to 2.75]) and routine health visit (UOR 3.63 [1.75 to 7.52]; AOR 3.75 [1.73 to 8.14]) were significant predictors of high outcomes of care.

Model 3 examined the influence of team processes on outcomes of care. High team processes (UOR 11.81 [95% CI 7.34 to 19.01]; AOR 14.92 [95% CI 8.11 to 27.44]) was a significant predictor of high outcomes of care. Also, high role clarity (UOR 5.36 [2.89 to 9.95]; AOR 3.02 [1.36 to 6.73]), living in an urban area (UOR 1.32 [0.90 to 1.95]; AOR 2.27 [1.31 to 3.95]), being employed (UOR 1.53 [1.02 to 2.29]; AOR 1.92 [1.10 to 3.36]), routine health visit (UOR 3.63 [1.75 to 7.52]; AOR 4.90 [1.96 to 12.22]) and patient as respondent (UOR 1.13 [0.74 to 1.71]; AOR 1.81 [1.02 to 3.22]) remained significant predictors of high outcomes of care.

**Fig. 2:**
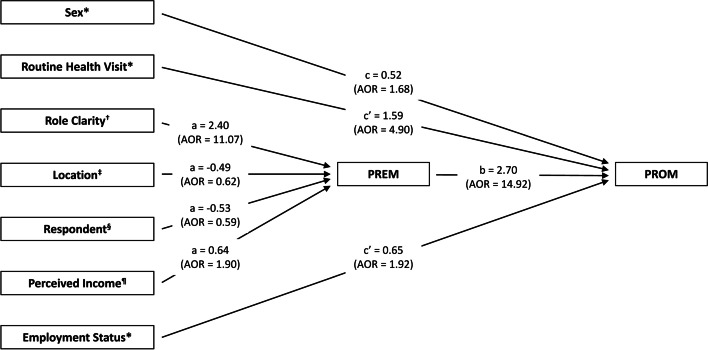
Indirect effects of the independent variables on outcomes of care mediated by team processes. * Significant direct effect on outcomes of care (all *p *< 0.05). † Significant coefficient of indirect effect of role clarity on outcomes of care (ab = 6.48; 95% CI [3.79 to 9.56]; *p *< 0.001). ‡ Significant coefficient of indirect effect of location on outcomes of care (ab = -1.32; 95% CI [-2.59 to -0.13]; *p *< 0.05). § Significant coefficient of indirect effect of patient as respondent on outcomes of care (ab = -1.43; 95% CI [-2.80 to -0.14]; *p *< 0.05). ¶ Significant coefficient of indirect effect of perceived income on outcomes of care (ab = 1.73; 95% CI [0.14 to 3.45]; *p *< 0.05). AOR = adjusted odds ratio; CI = confidence interval; PREM = patient-reported experience measure; PROM = patient-reported outcome measure

Moreover, the indirect effect of role clarity on outcomes of care (ab = 6.48) mediated by team processes was statistically significant (95% CI [3.79 to 9.56]; *p * < 0.001). Similarly, living in an urban area (ab = -1.32; 95% CI [-2.59 to -0.13]; *p *< 0.05), patient as respondent (ab = -1.43; 95% CI [-2.80 to -0.14]; *p *< 0.05), and income (ab = 1.73; 95% CI [0.14 to 3.45]; *p *< 0.05) were significant predictors of outcomes of care mediated by team processes. The results of the mediator effect of team processes are presented in Fig. 2.

## Discussion

We surveyed patients and families followed by primary healthcare teams with NPs across six sites in the Canadian province of Québec to examine their views of how these teams functioned. Most respondents had completed high school education. They perceived their health status to be good to excellent and their income to be adequate. Further, our research adds that in primary healthcare teams with NPs, team processes have a mediator influence on outcomes of care. Direct effects on outcomes of care were identified for individual characteristics (i.e., sex, employment status, routine health visit), whereas indirect effects were identified for individual characteristics (i.e., respondent, perceived adequacy of income) and organizational characteristics (i.e., location) and role clarity. Our study is the first study to measure PREMs and PROMs at the level of the healthcare team. Our results provide key insights on how primary healthcare teams with NPs contribute to outcomes of care, and highlight the importance of role clarity and the mediator effect of team processes on outcomes in teams with NPs while taking into account individual, team, and organizational characteristics.

Our study makes important empirical and conceptual contributions to the PREM and PROM literature by measuring the influence of role clarity on outcomes of care and the mediator role of team processes. Consistent with previous research [39, 40], characteristics related to the type of respondent, location, being employed, and a routine health visit were identified as significant predictors of perceived outcomes in teams with NPs. Globally, role clarity is a critical factor for success when implementing NP roles in teams [41–45]. The need for role clarity among team members has long been recognized as an imperative to optimize team functioning and outcomes from the provider perspective [8]. With its focus on roles and responsibilities, role clarity’s influence from the patient and family perspective had not yet been explicated in-depth [46]. Given the challenges identified by healthcare providers to ensure role clarity and the strong influence role clarity plays from the patient and family perspective, it is essential to monitor role clarity from the patient and family perspective and take steps to clarify roles among members of the healthcare team. The implementation of evidence-informed strategies to support role clarity and to develop non-technical skills, such as communication, is crucial when issues are identified. Interventions, including debriefing, structured communications, and speaking-up, show promise to improve team functioning [47]. Further, the co-development of an implementation plan with key stakeholders including patients, families, care providers, and decision-makers can support role clarification in teams [23].

Our findings have broad implications because of the wide-ranging use of teams in healthcare and the increased engagement of patients with multiple team members. As such, our study supports emerging evidence to link team functioning [48] and patient engagement [23] in primary care to performance. Moreover, our study provides insights into the team processes that contribute to effective team functioning and identifies team processes that can be improved to enhance team functioning and patient experience, and ultimately support greater patient engagement [39]. Determining what constitutes better team functioning from the patient and family perspectives is essential to improve care and give patients and families a voice in the organization of their healthcare [9]. More specifically, working closely with families to involve them as team members is an important strategy to enhance team processes and outcomes of care.

Furthermore, our study provides evidence of the mediator effect of team processes on outcomes of care, which can be seen to reduce the influence of other variables when included in the final model. Of note, patients and families expressed positive perceptions of team functioning and outcomes when healthcare teams included NPs. Such team processes, including decision-making, communication, care coordination, cohesion, problem-solving, a focus on the needs of patients and families, and trust are key components that patients and families appreciate and that can ultimately ensure a higher level of patient and family engagement. Outcomes, including timely care, dealing promptly with complications, and team knowledge and skill, were positively evaluated by patients and families. Although previous research has shown that healthcare teams that include NPs improve patient satisfaction with care [18, 40], our results explicate the micro-level processes in teams with NPs that contribute to patient experience and shed light on the mediator role played by team processes on outcomes at the team level.

### Implications for research and practice

Our study focussed on measuring perceived experience and outcomes of primary healthcare teams with NPs from the patient and family perspective. Based on our findings, we contend that paying close attention to role clarity and the quality of team processes, including communication, problem-solving, and a focus on patient and family needs represents a key strategy to improve patient primary care outcomes because these variables represent key modifiable factors. Similarly, providers in primary care and acute care teams report that teams function well when team members pay close attention to role clarity and team processes [19, 41].

In our study, team size, marital status, length of follow-up, and country of birth were not significant predictors of outcomes of care. Researchers have identified mixed findings when considering these characteristics [40, 49–51]. Further research is needed to take into account the mediator effect of team processes on outcomes of care while controlling for these factors. Additional condition-specific research is needed to determine the relationship between team processes, perceived outcomes, and condition-specific care outcomes for patients and families in different settings, including acute care, from the patient, as well as the provider perspective. The effects of these variables on patient and family engagement in their care, and other healthcare domains (i.e., teaching, administration, research) should also be investigated. Because we had identified a conceptual contribution, all the variables were retained in the statistical models, including non-significant effects identified in our sample. Subsequent research will need to further test the relationships in the conceptual framework and use a comparative study design to control more closely for confounding variables. Finally, complementary to our metrics approach to teamwork, a qualitative approach could be adopted to focus on tacit knowledge held by team members that could provide nuanced insights into inter-professional contingencies that also determine team functioning.

### Limitations

Even though our study included a small number of purposefully selected practices in one Canadian province, Québec, we anticipate that our findings are generalizable to teams in primary care across Québec and in other jurisdictions. At the outset, no group comparisons were planned and we did not control for NP characteristics, given that NP implementation in Québec was very recent at the time of data collection. Our response rate of 53% was slightly higher than response rates of 44 to 46% reported by Pieper et al. [52]. To further reduce the risk of bias in the assessment of team processes and outcomes, additional research is needed with teams that are selected at random, teams with and without NPs, teams with NPs who have different characteristics, with patients and families in home care and long-term care, and those of lower socio-economic status and lower educational levels. As previously stated, we decided a priori to not impute data in the analysis phase. Although our sample included 485 questionnaires with 16.7% missing data, it was sufficiently large, with almost 400 completed questionnaires. Furthermore, no question was left unanswered systematically, and the characteristics of patients and families who were excluded from the analysis because of missing data did not differ from those with which the analyses were carried out. Finally, although the use of categorical response options in the questionnaire limits our ability to apply more sophisticated statistical techniques aimed at reducing the percentage of missing data in the analysis [53], it eases the response burden for participants.

## Conclusion

We completed a cross-sectional survey of patients and families in Québec, Canada, under the care of primary healthcare teams with NPs to examine patient and family perceptions of team functioning and outcomes of care. We found that team processes exerted a mediator influence on outcomes of care, and role clarity was the only variable to influence team processes and outcomes of care. Although different patient and organizational characteristics influenced either team processes or outcomes of care, additional research is needed in teams without NPs, selected at random, and in different settings, to further explicate and test the relationships between patient, team and organizational characteristics, role clarity, team processes, and outcomes of care that we identified in our study. Such knowledge will support the creation of high functioning healthcare teams from the perspective of patients, families, healthcare providers, and decision-makers.

## Data Availability

The datasets used and/or analysed during the current study are available from the corresponding author Kelley Kilpatrick on reasonable request.

## Abbreviations

AOR: Adjusted odds ratio
BE: Beliefs about team effectiveness
CI: Confidence interval
DV: Dependent variable
IV: Independent variable
NP: Nurse practitioner
PREM: Patient-reported experience measure
PROM: Patient-reported outcome measure
PTE: Perceptions of team effectiveness
rs: Rho coefficients
SD: Standard deviation
UOR: Unadjusted odds ratio
